# The Effectiveness of an Exercise Program Based on Motor Learning Principles for the Correction of the Forward Head Posture: A Randomized Controlled Trial

**DOI:** 10.3390/brainsci15080873

**Published:** 2025-08-15

**Authors:** Stephani Argyrou, Pavlos Kitixis, Zacharias Dimitriadis, Anna Christakou, Nikolaos Strimpakos, George Paras, Maria Tsioutsoumaka, Eleni Kapreli

**Affiliations:** 1 Clinical Exercise Physiology and Rehabilitation Research Laboratory, Department of Physiotherapy, University of Thessaly, 35132 Lamia, Greece; konstef@msn.com (S.A.); mariatsiou16@yahoo.gr (M.T.); ekapreli@uth.gr (E.K.); 2 Human Performance and Rehabilitation Laboratory, Department of Physiotherapy, University of Thessaly, 35132 Lamia, Greece; pkitixis@uth.gr (P.K.); gparas@uth.gr (G.P.); 3 Health Assessment and Quality of Life Research Laboratory, Department of Physiotherapy, University of Thessaly, 35132 Lamia, Greece; zdimitriadis@uth.gr (Z.D.); nikstrimp@uth.gr (N.S.); 4 Laboratory of Biomechanics, Department of Physiotherapy, School of Health Sciences, University of Peloponnese, 23100 Sparta, Greece

**Keywords:** craniovertebral angle, forward head posture, motor learning, rehabilitation

## Abstract

**Background/Objectives:** The aim of this study was to examine the effectiveness of an exercise program based on principles of motor learning with cognitive elements (such as attention) in the correction of Forward Head Posture (FHP). **Methods**: A total of 55 university students from the University of Thessaly, aged between 18 and 25 years, participated in this study. Volunteers found to have a craniovertebral angle <50° were randomly divided into two groups: the intervention group IG (*n* = 27) and the control group CG (n = 28). The IG followed a treatment protocol consisting of three 30–45-min sessions per week for four weeks, a total of twelve sessions, while the control group received the same content instructions and group sessions upon completion. **Results:** After the four-week intervention period, significant improvements were observed in both static and dynamic craniovertebral angle measurements (*p* < 0.05) when comparing the IG to the CG. Additionally, there was a notable increase in the endurance of the deep neck flexors (*p* < 0.05), even though the intervention for the IG did not include specific strength exercises. Furthermore, participants with FHP were able to transfer (B–C) motor skills (*p* < 0.01) acquired during static position tasks to a similar but dynamically untrained task. They also retained (B–D) improvements in posture and strength during the two-week detraining period (*p* < 0.01), indicating sustained motor learning effects. **Conclusions**: The exercise intervention was successful at decreasing FHP in subjects, sustaining the results for a two-week period. This study supports the effectiveness of postural training by a program based on motor learning principles. New rehabilitation strategies based on motor control and motor learning could be introduced into physiotherapy practice to increase effectiveness.

## 1. Introduction

Postural control, with minimal muscle tension and strain, is a key element of maintaining musculoskeletal health, preventing dysfunction and pain [1]. Studies show that poor posture, such as forward head posture (FHP) and protracted shoulders, often develops during adolescence and can lead to neck pain, shoulder pain, and tension headaches [2,3]. Although there are raising arguments for the clinical value of postural reeducation [4], studies showed that it can significantly reduce pain and improve symptoms, particularly in spinal disorders [5,6,7].

FHP is characterized by the head being positioned forward with hyperextension of the cervical spine, causing shortening of the upper trapezius, cervical extensor muscles (suboccipitals, spinalis, splenius), sternocleidomastoid, and levator scapulae. This leads to a discrepancy in the length–tension relationship and decreased muscle strength [8]. In addition, people with FHP develop poor orthostatic control by shifting the center of gravity and impairing fine motor control [9]. Unhealthy motor patterns due to cervical spine posture can lead to overall body balance disorders [10], neck muscle imbalances, chronic neck pain [11,12], and even respiratory muscle weakness or breathing pattern dysfunction [13,14,15].

The increasing prevalence of FHP in modern society, primarily driven by prolonged use of digital devices, underscores the need for early intervention [16]. Correcting FHP offers numerous health benefits, particularly in alleviating musculoskeletal strain and improving overall body function. By realigning the head and neck, the excessive load on the cervical spine is reduced, which helps decrease chronic neck pain, muscle tension, and the risk of cervical degeneration [17]. Addressing FHP through targeted exercises can mitigate its detrimental effects on musculoskeletal function, respiratory health, and overall quality of life [18].

Despite researchers’ efforts to develop effective treatments for long-term correction of FHP—including strength training, stretching, endurance training, movement control, and proprioceptive exercises—little evidence supports the success of these interventions [19,20]. For example, a systematic review and meta-analysis by Nazwar et al. [21] reported that stretching and strengthening interventions led to an average craniovertebral angle improvement, although retention levels were inconsistently reported across studies. Motor learning-based interventions use external attentional focus, task-specific training, and feedback mechanisms (e.g., visual, proprioceptive), potentially leading to better neuromuscular adaptations [22]. Consequently, there is a growing interest in musculoskeletal rehabilitation programs using means that incorporate motor control and learning principles for better results and long-term retention. Strategies such as external attentional focus and cognitive elements (attention, motivation, feedback, awareness) have been suggested as more effective [23,24], but their impact on FHP reeducation has not yet been examined. Even more, there is a knowledge gap in understanding how motor learning strategies can be specifically applied to FHP reeducation, and whether such interventions lead to better retention and transfer of postural improvements.

Previous studies have demonstrated the effect of motor control and motor learning interventions in managing neck pain and improving cervical function. For instance, Tsiringakis et al. [25] found that motor control training of deep neck flexors using pressure biofeedback significantly reduced pain and disability in individuals with neck pain. Similarly, Martin-Gomez et al. [26] reported that craniocervical flexion exercises were more effective than other treatments for non-specific chronic neck pain in improving motor function and reducing symptoms. Interestingly, another randomized controlled trial highlighted that motor control training targeting areas beyond the cervical region, such as lumbar motor control exercises, has also shown complementary benefits in patients with chronic neck pain [27].

Posture is influenced not only by physical factors but also by mental functions and social behaviors. The interplay between these cognitive and behavioral dimensions in exercise-based interventions has been shown to improve and support long-term maintenance, yet these are not consistently addressed in standard practice [28]. Recent evidence suggests that emotional states and mental health issues, such as anger, anxiety, stress, depression, and overall self-esteem, may influence posture [29,30], as well as total fatigue, whether muscular or mental. Despite these findings, there is a lack of comprehensive research integrating psychosocial factors into FHP rehabilitation programs, which raises concerns about the validity and applicability of current treatment outcomes.

Moreover, assessing motor performance alone is insufficient to measure motor learning and neuroplastic changes. Motor learning is assessed through three key elements: acquisition, retention, and transfer of skills [31]. A skill is considered truly learned only when retention and/or transfer is demonstrated. However, studies on FHP correction programs typically focus solely on motor performance outcomes [32,33], with only a few including retention measurement [34,35]. There remains a need for clinically grounded studies that assess the acquisition, retention, and transfer of motor skills related to FHP correction, to better understand long-term functional improvements and real-world applicability.

Motor control and learning principles are foundational in clinical rehabilitation, particularly in the treatment of patients with neurological or musculoskeletal impairments [36]. The concepts of neuroplasticity, task-specific training, and feedback-based motor learning play critical roles in recovery [37]. Neuroplasticity facilitates the adaptation of the central nervous system to therapeutic interventions, enabling the retraining of motor control systems for improved head alignment [38]. Task-specific training, which incorporates functional movements and postural awareness, helps in realigning the head and neck by engaging and strengthening relevant muscle groups, thereby addressing FHP more effectively [19,39].

Additionally, understanding motor synergies is vital for correcting FHP, as abnormal synergies often involve overactivity in the upper trapezius and levator scapulae, alongside underactivity in the deep cervical flexors [40]. Interventions that target these specific muscle imbalances—through strengthening and stretching exercises—can help restore proper posture. Feedback mechanisms, including visual and proprioceptive cues, further enhance the effectiveness of these interventions by promoting correct postural habits and preventing recurrence [41]. Collectively, these motor control principles form therapeutic strategies that could progressively improve postural alignment and alleviate the symptoms associated with FHP.

Training to correct forward head posture may lower the cervical dysfunction-related risk factors or decrease symptoms. However, given the evidence of potential benefits of head posture reeducation, little is known about whether the modification is trainable in clinical physical therapy practice.

Therefore, the aim of this study was to investigate the effectiveness of a clinically feasible, motor learning-based exercise program in improving FHP through static and functional tasks during a progressive four-week training. This study addresses an important knowledge gap by integrating motor learning principles into FHP rehabilitation and assessing both the short-term effects and retention following a structured training and detraining period.

## 2. Materials and Methods

### 2.1. Study Design

This single-blind randomized controlled trial (RCT) was conducted in accordance with the Consolidated Standards of Reporting Trials (CONSORT) guidelines (Table S1) [42]. The study complied with the Declaration of Helsinki (DoH)—Ethical Principles for Medical Research Involving Human Participants—and its latest amendments. This trial was registered with ClinicalTrials.gov (Identifier: NCT03006497), a service of the U.S. National Institutes of Health.

### 2.2. Participants

Participants were conveniently recruited from the Department of Physiotherapy in Lamia, Greece. Prior to participation, all volunteers signed an informed consent form. Sample size was determined using G*Power software (version 3.1.9.3), which indicated that a minimum of 26 participants per group was required (two groups, α = 0.05, power = 80%, effect size f = 0.4) [43].

The inclusion criteria specified students aged 18–26 years who exhibited anterior head posture with a craniovertebral angle of less than 50°. Participants’ head posture was assessed using lateral photographs [44]. Exclusion criteria included a history of cervical spine injury or surgery, neurological, musculoskeletal, mental, or visual disorders, medication use, neck pain, recent physical therapy for cervical dysfunction within the last six months, changes in physical activity during the study period, and a BMI greater than 30.

Out of 149 volunteers initially screened, 67 met the eligibility criteria. Twelve declined to participate, leaving 55 volunteers who were randomly assigned to either the intervention group (n = 27) or the control group (n = 28). Participants were randomly assigned to groups using a computer-generated random sequence (https://www.random.org; accessed on 20 October 2016), and allocation was concealed using opaque, sealed envelopes by an independent researcher.

### 2.3. Interventions

Volunteers in the intervention group participated in a motor control therapeutic exercise (MCTE) program conducted over four weeks, with three sessions per week, each lasting 30–45 min. The training protocol incorporated simple functional activities (Table 1) and was divided into two progressive parts, each targeting postural alignment, stability, and sensorimotor control (see Appendix A).

**Figure 1:**
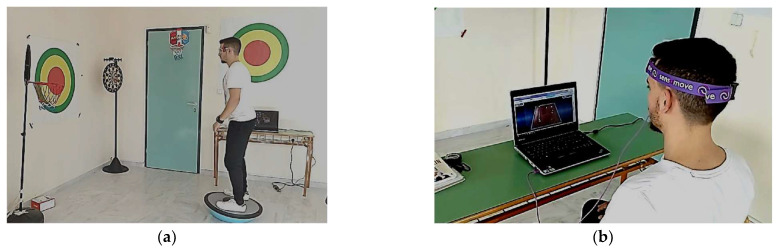
Exercise program based on principles of motor learning with cognitive elements: (**a**) Attention, motivation, feedback; (**b**) Reasoning and video gaming.

The first part consisted of static and dynamic exercises performed in different positions (e.g., seated or standing). Exercises involved manipulation of objects (e.g., weights), balance testing with unstable surfaces (e.g., platforms, medicine balls), and movements in varying conditions, such as lateral stepping with or without shoes. Postural alignment was supervised through aiming at bound targets on a wall, with a laser device mounted on the head, promoting external focus. Progression aligned with Gentile’s two-dimensional taxonomy and was based on increasing complexity of a task (e.g., surface instability or dual-task elements) [45,46]. Auditory feedback was given using a metronome to guide timing and rhythm (Figure 1a).

The second part involved virtual reality-based tasks using a 3D Cervical Trainer device (Sensamove, Groessen, The Netherlands) connected via Bluetooth to a laptop with 3D Cervical Trainer software (www.sensamove.com; accessed on 25 October 2016) [47]. In detail, the Cervical Trainer was attached to the participant’s head (Figure 1b) [48]. Participants performed head and body movements in both seated and upright positions. The goal was to maintain correct neck and shoulder posture as well as the vertical alignment of the body (spine, pelvis, knees, and ankles) in response to visual tasks (e.g., game targets). The exercises progressed based on Gentile’s two-dimensional classification table and focused on external attentional cues. External feedback has been demonstrated to enhance the automatization of the learning process for skill control, thereby leading to improved acquisition and execution of the skill [49].

Following the program, there was a two-week detraining period without any intervention. This interval was chosen as prior studies show that motor performance is well retained over eight days in human skills, but declines significantly by 14 days in animal motor learning tasks, supporting its appropriateness for assessing short-term retention [50,51].

The control group did not engage in any exercise during the study. At the conclusion of all measurements and upon confirming the effectiveness of the proposed intervention, instructions were provided to the control group participants, who subsequently attended group sessions containing the same components as the intervention program.

### 2.4. Outcome Measures

The primary outcome measures were the FHP during standing (static FHP) and walking (dynamic FHP). Secondary outcomes measures were the endurance of deep neck flexors, self-esteem, mood, physical fatigue, mental fatigue, attention, and discomfort (head, neck, shoulders and upper arms, middle back, low back, forearms, and wrist/hand areas).

#### 2.4.1. Static Forward Head Posture

Static FHP was assessed by capturing three standardized lateral photographs of each participant’s head position while standing. Participants stood barefoot on the side, in a relaxed position, and with their eyes looking straight ahead. A 24.2 MP DSLR camera (D3300, Nikon Corporation, Tokyo, Japan) was positioned on a tripod 1.5 m away at shoulder height and perpendicular to the sagittal plane.

**Figure 2:**
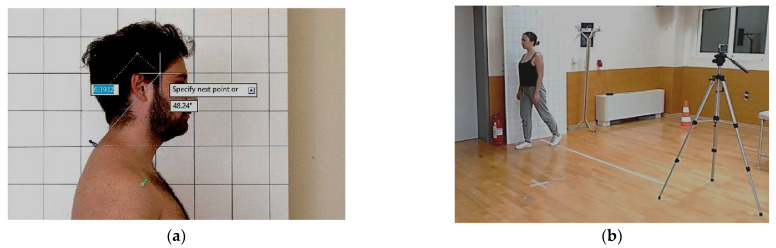
Measurement of craniovertebral angle: (**a**) Static FHP; (**b**) Dynamic FHP.

The craniovertebral angle, which serves as the representative measure of FHP, was defined as the angle between a horizontal line through the spinous process of C7 and a line connecting C7 to the tragus of the ear (Figure 2a). C7 was identified via palpation during cervical flexion and extension, following the procedure described by Dimitriadis et al. [36], and marked with a Dermatograph pencil prior to imaging. Angle measurements were calculated in AutoCAD software from the average of the three trials recorded [44].

#### 2.4.2. Dynamic Forward Head Posture

Dynamic FHP was assessed during walking of a predetermined 5-m distance. Participants were asked to walk at a comfortable pace and step with their left foot on a marked white line positioned in front of a square frame. Three gait trials were captured from a sagittal view using an iPhone 6 mounted on a tripod in slow-motion video mode (720p, 240 fps). Frames were extracted during midstance, defined as the moment when the foot is flat on the ground and the contralateral side is off the ground (Figure 2b).

The same anatomical landmarks (tragus and C7) were pre-marked as in the static condition. The angle calculation was performed using Kinovea software (v0.8.15) and then verified with AutoCAD software (version R21.0). Laboratory investigations have shown this procedure to be highly reliable (ICC = 0.90–0.92, SEM = 1.44–1.84°, SDD% = 8.2–10.5) [52].

#### 2.4.3. Endurance of Deep Neck Flexors

Endurance of deep neck flexors were examined with the craniocervical flexion test (CFT). The test was performed according to the instructions provided by Broisler et al. [53]. During this test, participants lay supine with their knees bent and the cervical spine in neutral. A pressure biofeedback unit (Chattanooga Stabilizer, DJO LLC, Tennessee, USA) is positioned under the participant’s occiput in supine and inflated to a baseline of 20 mmHg. Each participant was asked to perform and maintain a movement of craniocervical flexion at five different pressure levels (22, 24, 26, 28, and 30 mmHg). Endurance was measured with two methods: the maximal pressure at which the participant could maintain the craniocervical flexion for three tries of 10-sec hold without any other compensatory strategy (e.g., sternocleidomastoid activation). The craniocervical flexion test has been validated and is highly reliable (ICC > 0.98, SEM = 8.94 mmHg, SDD%= 24.7) [54].

#### 2.4.4. Self-Reported Questionnaires

The degree of discomfort was assessed in seven anatomical regions (head, neck, shoulders and upper arms, middle back, low back, forearms and wrists/hands) by using a body chart and a visual analog scale (VAS: 0–10) [55]. The “Body Chart” utilized was a modified version of the Cornell Musculoskeletal Discomfort Questionnaire (CMDQ), based on previous studies [56].

Participants’ self-esteem was evaluated using the Rosenberg Sorensen Self-Esteem Scale (RSES), a 10-item instrument with a four-point Likert scale, which has been cross-culturally validated in Greek [57]. Mood and fatigue levels were measured using a 10-point numeric rating scale (0 = worst, 10 = best). Additionally, attention levels in the intervention group were assessed through the adapted 10-item Attention Questionnaire of Rehabilitated Athletes Returning to Competition. Questions such as “I was concentrated on my goal” were rated on a five-point Likert scale and measured the ability of selective attention after rehabilitation [58].

### 2.5. Procedure

**Figure 3:**
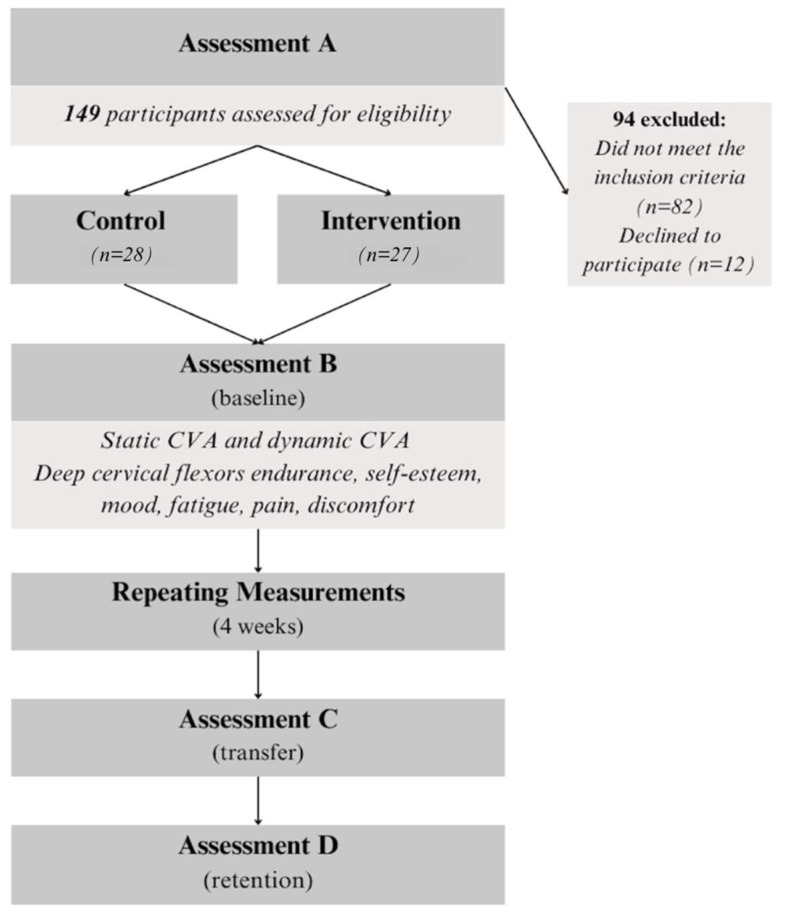
Study flow chart.

These variables were assessed at baseline (assessment B), at the end of the four-week training period (assessment C), and after the two-week detraining period (assessment D). In order to obtain performance curves, static and dynamic FHP, as well as self-esteem, mood, physical fatigue, and mental fatigue, were additionally assessed 12 times during the training period. Attention was additionally assessed after each training session as an indication of the subjects’ participation level during the program. (Figure 3). All the assessments were performed by an appropriately trained senior postgraduate physiotherapist blinded to the procedure. Participants and therapists could not be blinded due to the nature of the intervention.

### 2.6. Data Analysis

Normality of data was assessed with the Kolmogorov-Smirnov test. Descriptive statistics were presented as means (for central tendency) and standard deviations (as measures of dispersion). The effectiveness of intervention (baseline B, measurement C, measurement D) was evaluated over time using two-way mixed and repeated measures Analysis of Variance (ANOVA) and examining between and within group interactions. Additionally, post hoc comparisons were conducted to further investigate change scores from specific timepoints (baseline–C, C–D, and baseline–D differences) between groups. Pearson correlation coefficients (r) were used for examining correlations between craniovertebral angle and fatigue, mood, and self-esteem. Significance level was set at α = 0.05. All data analyses were conducted using the Statistical Package for Social Sciences (SPSS), version 27.0.

## 3. Results

Baseline characteristics did not differ significantly between the two groups (*p* > 0.05) (Table 2). All variables were found to be normally distributed across groups and time points, as indicated by non-significant Kolmogorov–Smirnov test results (*p* > 0.05). Three participants (5.5%) withdrew during the study, one participant from the intervention group and two from the control group, and they were not included in the final analysis.

### 3.1. Static and Dynamic FHP

**Figure 4:**
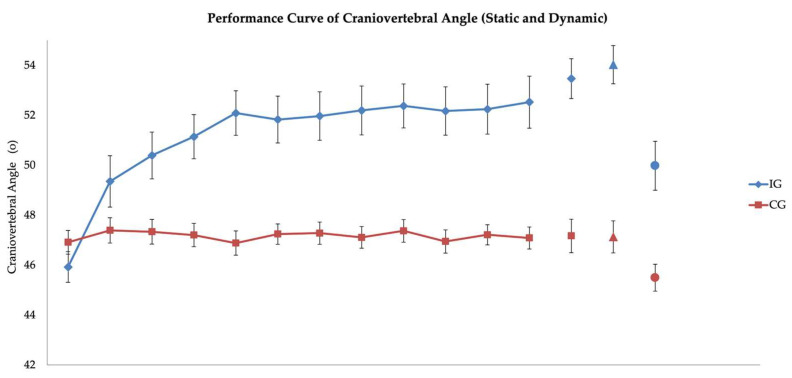
Performance curve repeated measurements: craniovertebral angle static (■), transfer measurement—craniovertebral angle dynamic (•), and retention measurement (▲) in both groups. Bars represent the standard error of measurement (SEM).

A significant group × time interaction was detected for both static FHP (F(1.4, 70.6) = 124.330, *p* < 0.001) and dynamic FHP (F(1.1, 59.6) = 28.394, *p* < 0.001) using mixed ANOVA (Figure 4). To account for baseline imbalances between groups, an analysis of covariance (ANCOVA) was conducted using baseline craniovertebral angle as a covariate. The results revealed statistically significant differences post-intervention between groups in both static and dynamic craniovertebral angle after controlling for baseline values (*p* < 0.001).

**Figure 5:**
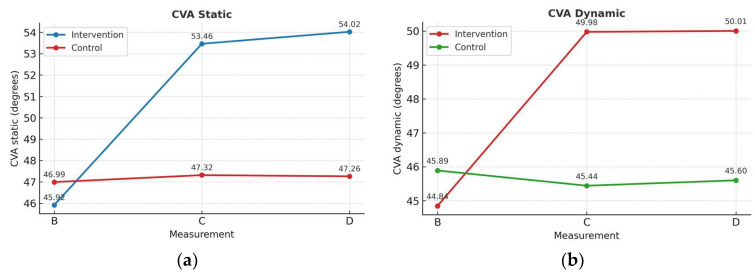
Comparison means between groups of (**a**) Static; (**b**) Dynamic craniovertebral angle (CVA) in the periods baseline (B), post-intervention (C), and retention (D).

Post hoc comparisons with Bonferroni correction revealed that the IG demonstrated significant improvements in static craniovertebral angle from baseline (B) to post-intervention (C) (MD = 7.54°, 95% CI [6.37, 8.71], *p* < 0.001), and from baseline to retention (D) (MD = 8.10°, 95% CI [6.80, 9.40], *p* < 0.001). No significant difference was observed between timepoints C and D (*p* = 0.264), indicating maintenance of improvements (Figure 5a). Similarly, IG showed significant changes in dynamic FHP from timepoints B to C (MD = 5.13°, 95% CI [3.04, 7.22], *p* < 0.001) and from B to D (MD = 5.16°, 95% CI [2.70, 7.62], *p* < 0.001), with no significant change between C and D (*p* > 0.05) (Figure 5b) (Table 3).

In contrast, the CG depicted no significant changes in static or dynamic FHP across all timepoints (*p* > 0.05), indicating that the observed improvements in IG were not attributable to external factors such as time or repeated testing.

### 3.2. Deep Neck Flexors Endurance

ANOVA indicated a significant group × time interaction for endurance of deep neck flexors examined with the craniocervical flexion test (F(1.5, 75) = 16.356, *p* < 0.001) (Table 3). Post hoc analysis revealed statistically significant changes in endurance of deep neck flexors in the IG across all time periods (*p* < 0.01), compared to CG, which demonstrated no significant change across the same time points (Figure 5).

### 3.3. Self-Reported Outcomes

There were no significant changes in discomfort over time (*p* > 0.05). A significant correlation was found between dynamic FHP and fatigue for the control group at C and D measurements (*p* < 0.05, r = 0.034–0.045). However, correlations between head posture (static and dynamic) and self-reported outcomes (fatigue, mood, self-esteem) were not significant (*p* > 0.05) for other measures.

Participants in the IG showed a gradual increase in attention levels across sessions following intervention (Figure 5). After further calculation, repeated measures ANOVA indicated a non-significant session*phase interaction (F = 1.59, *p* > 0.05), indicating that attention levels remained relatively stable when compared with baseline measurements.

## 4. Discussion

This study provides evidence that confirms the intended aim regarding the effects of MCTE on posture correction measured in the cervical region in asymptomatic subjects. The results of this study demonstrated that a four-week retraining program with principles of motor learning and motor control, combined with cognitive features such as attention, motivation, and feedback, resulted in a posture improvement, with an increase in the craniovertebral angle and endurance of the deep cervical flexor muscles. In detail, between-group comparisons showed a statistically significant difference in measures C and D. This suggests that the MCTE program significantly improved these measures, and the improvement was maintained at the end of the program. Additionally, it appeared that the dynamic craniovertebral angle generally has lower values than the static craniovertebral angle across all measurement periods.

IG showed a 10.9% improvement (50.39 ± 4.77) in the repeated measurements of static craniovertebral angle from the first week. However, performance values during the first week showed variance, which is typical in the initial cognitive stage of skill acquisition [31]. High scores are more likely to occur due to random factors than a true understanding of the skill. Craniovertebral angle continued to increase in the following weeks (2nd and 3rd), by 11.2% (51.82 ± 4.79) and 11.4% (52.37 ± 4.49), respectively. IG’s performance curve depicted a negative trend, with a large initial increase in performance followed by a decreasing rate of improvement at later stages. CG measurements depicted no significant change throughout the same period, supporting the effectiveness of the intervention (Figure 5).

Moreover, transferability of training effects was present beyond static posture. The dynamic craniovertebral angle, which was recorded during gait, presented an increase in the IG between the B (45.89 degrees) and C measurements (49.98 degrees). After two weeks of detraining, the craniovertebral angle was maintained at 50 degrees (measurement D). These results are clinically significant as the normal mean craniovertebral angle is considered to be equal to or higher than 50°, with a range of values (static craniovertebral angle) between 55.02 ± 2.86° [41,59]. Although no standardized values for dynamic craniovertebral angle exist, our findings provide preliminary evidence that motor patterns can transfer to functional tasks (e.g., gait), providing a significant implication for real-world applicability.

Previous research has demonstrated clinically meaningful improvements in FHP using different intervention techniques, ranging from four to eight weeks. A study of 60 participants concluded that a corrective program involving postural control exercises can significantly improve the static craniovertebral angle by 4.2° [40]. Abd El-Azeim et al. [60] reported a mean craniovertebral angle increase of 6.2° over six weeks when scapular stabilization was added to postural correction exercises. Mylonas et al. [61] found a 5.6° craniovertebral angle increase and a 4.9-point reduction in neck disability index after four weeks of combined soft tissue mobilization and neuromuscular training. Other researchers used technological advancements (smartphones) to promote postural re-education, which not only resulted in a 6.7° craniovertebral angle increase but also improved deep neck flexor endurance by 2.8 mmHg after eight weeks [7]. In comparison, our study demonstrated a 7.5° improvement in static and a 5.1° improvement in dynamic craniovertebral angle, suggesting that structured motor learning strategies may also be effective for postural correction, and adding a functional perspective (dynamic craniovertebral angle) as an outcome.

**Figure 6:**
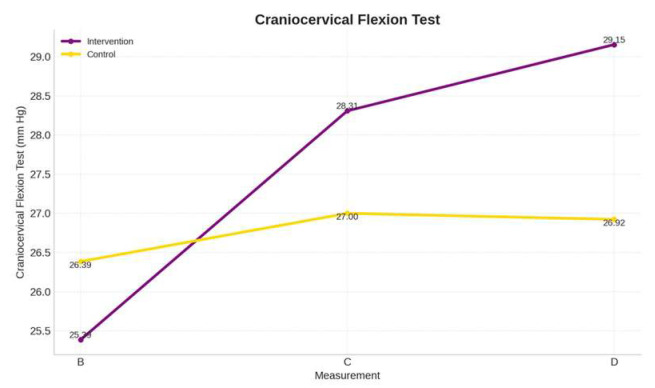
Comparison means between groups of the Craniocervical Flexion Test (CFT) in baseline (B), post-intervention (C), and retention (D) timepoints.

Regarding the perception of endurance as measured by the CFT, there was an increase in this outcome, even though the program did not include strengthening exercises. In measure C, the increase was 2.92 mmHg, reaching 28.30 mmHg. For measure D, the results showed 29.15 mmHg. In contrast, the control group values ranged from 26–27 mmHg (a low rate). All changes from period B to C were statistically significant only for the intervention group (Figure 6). These results are clinically important, as the normal range is assumed to be between 28–30 mmHg, suggesting that improved motor control and postural awareness alone can yield functional strength gains. In the study of Heydari et al. [62], it was revealed that the minimally clinically important difference for the craniovertebral angle of people with FHP is at least 1.40 degrees. This reinforces the idea that motor learning strategies may serve as an alternative or complement to traditional strengthening approaches [54,63].

While the intervention led to statistically significant improvements in both dynamic craniovertebral angle and neck flexor endurance (CFT), the effect sizes (r = 0.34 and r = 0.32, respectively) suggest that these changes may be moderate rather than substantial. Not all statistically significant changes necessarily translate to meaningful functional benefits. In other words, although the results are encouraging, future research could address the potential for refining the intervention, such as adjusting the training duration, intensity, or individualization, to achieve greater benefits.

Previous studies on neck flexor endurance in individuals with FHP have shown comparable gains with interventions focusing on proprioception or neuromuscular training. Abadiyan et al. [7] reported a 2.8 mmHg increase in deep neck flexor endurance after eight weeks of global postural re-education supplemented by a smartphone app. Similarly, Khosrokiani et al. [27] found a 3.4 mmHg improvement in endurance following an eight-week lumbar motor control training program, highlighting the potential for cross-regional effects on cervical function. These findings align with our results, where a 3.8 mmHg increase was observed, suggesting that targeted motor learning strategies may yield comparable or even superior outcomes within shorter durations.

Discomfort, as reported on the Body Chart, suggested a non-significant reduction between groups over various time periods. This lack of significant findings may be attributed to the sample’s demographics (relatively young age of the participants), who were less likely to present musculoskeletal disorders such as deformities or degenerative conditions. However, our findings are consistent with prior studies indicating a weak correlation between perceived discomfort and cervical posture in healthy populations [61].

**Figure 7:**
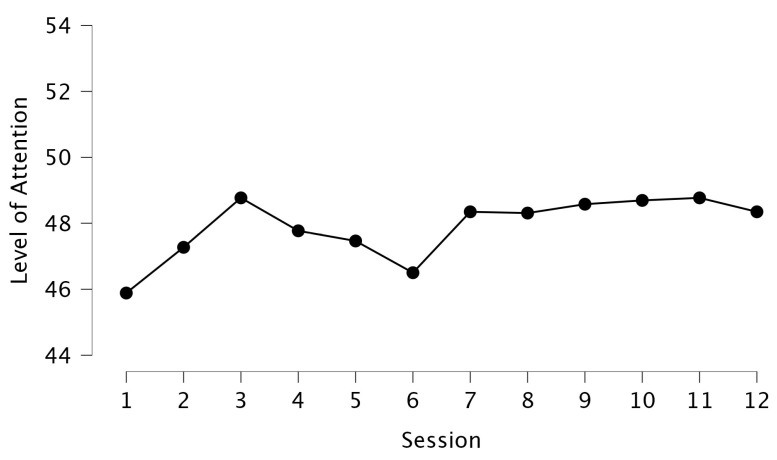
Level of attention as depicted per session in the intervention group (IG).

Cognitive engagement and attentional focus were maintained throughout the intervention, reflected by scores in the attention test. In the first session, the average value was 45.35 ± 7.48, whereas the last session corresponded to 48.35 ± 4.76. From the diagram (Figure 7), we observe a small plateau from the end of the 2nd to the beginning of the 3rd week. This observation comes to agreement with the performance curve of the volunteers and probably justifies it stability of performance observed at this stage as a result of the normalization of movements and the transition of the stages of memory [45]. Minimal dropout rate from the IG (1.37%) further suggests high participant engagement, likely due to the program’s cognitive demands and feedback mechanisms.

Although previous studies have examined changes in outcomes regarding the cervical position, endurance, and pain, there is no evidence examining motor learning principles or motor control post-intervention effects (retention and transfer), to support clinical effectiveness. In addition, most available intervention programs focus on strength and stretching exercises, with durations ranging from over 32 weeks to as short as six weeks [59]. Precise gains vary across studies, although long-term maintenance and progression parameters remain a challenge. In the current study, Gentile’s taxonomy guided the progression of tasks from static to more dynamic conditions (motor learning). This structured task progression supported cognitive and motor challenges (motor performance) appropriate to each stage of learning (acquisition, retention, and transfer), adhering to motor learning principles within a clinically feasible format [31,64]. Participants with FHP were able to transfer the motor skills acquired during static position tasks to a similar but dynamically untrained task, such as walking. They also maintained perceived improvements in posture and strength during the detraining period, indicating sustained motor learning effects (Table 3).

### 4.1. Limitations

This study has several limitations that must be acknowledged. First, the sample was limited to young adults with poor posture, which may not fully represent individuals with chronic neck pain or other pathological conditions. Thus, the results may not be directly generalizable to clinical populations with diagnosed neck pain. Second, there were a number of dropouts, which could have impacted the overall findings and introduced a potential bias. A third limitation is the lack of exploration into whether postural correction impacts not only neck pain symptoms but also other disorders, such as breathing or anatomical dysfunctions, which may be relevant in broader clinical contexts. Fourth, the study’s intervention was constrained by time and frequency parameters that might not have been sufficient to observe long-term effects.

### 4.2. Clinical Applicability

The findings from this study highlight the importance of integrating motor learning principles to provide rehabilitation interventions and improve parameters such as the craniovertebral angle and cervical flexor muscle endurance in people with asymptomatic FHP. The present protocol, with MCTE based on Gentile’s taxonomy, demonstrates the potential for applicability in rehabilitation contexts for early postural correction. This is particularly relevant for younger populations who exhibit FHP due to extended periods of digital device usage (such as smartphones). It provides a feasible implementation without the need for specialized equipment. Its emphasis on external focus, feedback, and progressive task difficulty could be integrated into preventative instructions to address postural dysfunction. Inclusion of both static and dynamic craniovertebral angle assessments strengthens the functional relevance of the findings, with improvements demonstrating transfer to real-world tasks such as gait. Relatively short intervention duration (four weeks) and a demonstrated retention of postural improvements after detraining suggest that this approach may be both time-efficient and sustainable.

### 4.3. Recommendations for Future Research

Future research could focus on a larger sample size, longer follow-up periods (such as two months post-intervention), and address skill attrition during exercise breaks to better evaluate the sustainability of outcomes and strengthen their clinical relevance in a rehabilitation setting. Researchers should also consider investigating postural improvements in older adults or clinical populations, such as patients with chronic neck pain, postural instability, or spine disorders. Innovative solutions such as mobile applications, home exercise programs, and cognitive education would promote continuity and ensure long-term retention of intervention benefits.

## 5. Conclusions

This study demonstrated that a simple, targeted exercise program based on motor learning principles and cognitive training can improve postural alignment in young adults. Administered three times a week over four weeks, the intervention resulted in significant increases in both static and dynamic craniovertebral angle and cervical flexor endurance. Notably, the program results were not only retained after a two-week detraining period but were also transferred to more functional conditions (demanding) such as walking, indicating real-world applicability. These findings support the potential of incorporating motor learning strategies into standard physiotherapy protocols for postural correction. The intervention’s ability to produce retained and transferable improvements highlights its potential as a cost- and time-effective strategy to enhance rehabilitation outcomes. Future research could evaluate its application in broader age groups and clinical populations.

## Figures and Tables

**Table 1:** Motor control exercises used during the training program. All the exercises have the same goal: to hold the laser target.

	Action Function (Body Stability)	
Environmental Context	No Object Manipulation	Object Manipulation
Stationary Regulatory Conditions and No Intertrial Variability	**1^A^** Sitting position with laser, viewing a video/reading/playing 3D games. Progressively ↓ **1^a^** Standing position, lateral and up-down steps /playing 3D games.	**1^Β^** Sitting position with laser: writing a text with PC. Standing position: playing with a toy or a ball/holding weights and playing 3D games. Progressively ↓ **1^b^** Standing position: holding weights (different kilograms) and lateral and up-down steps.
Stationary Regulatory Conditions and Intertrial Variability	**2^A^** Standing on different surfaces (mattress, flywheel, wood, carpet, etc.). Sitting in different chairs (hard, anatomic, high, low, stool). Progressively ↓ **2^a^** Steps in place, sideways and backwards on different surfaces, with shoes and without. Sitting on different chairs and medicine ball (unstable) with laser viewing a video/reading/playing 3D games.	**2^Β^** Standing, pitching, or sitting on different unstable surfaces (platforms, BOSU ball, medicine ball) and holding a weight/throwing or beating a basketball/THERABAND exercise. Progressively ↓ **2^b^** Sitting on different unstable surfaces, balance disturbance and ball bounce, or shooting basketball free throws.
In-Motion Regulatory Conditions and No Intertrial Variability	**3^A^** Steady steps in a seated position with a metronome (fixed speed and weight). Progressively ↓ **3^a^** Sitting on a medicine ball, field steps with a metronome, or standing-position steps in place with a metronome, increasing the height of the step.	**3^Β^** Holding a ball or weight and sitting on a medicine ball, field steps with a metronome, or standing position steps in place with a metronome and increasing the height of the step. Progressively ↓ **3^b^** Sitting on a medicine ball/Step and shooting a basketball with constant speed and distance/ flying darts at a target.
In-Motion Regulatory Conditions and Intertrial Variability	**4^A^** Steady steps in a seated position with a metronome (different speeds and weights on the legs). Progressively ↓ **4^a^** Sitting on a medicine ball and balance disturbance, shooting a basketball on command and from different distances.	**4^Β^** Standing on a platform (BOSU ball), balance disturbance, shooting a basketball on command. Progressively ↓ **4^b^** Sitting on a medicine ball, balance disturbance, handling a football with the legs or basketball with the hands, and the same exercises on a standing position on a platform.

“↓” indicates that the difficulty increases as it progresses downward in the Table.

**Table 2:** Baseline characteristics of participants (nine male and 17 female participants for both groups).

	Control Group (*n*=26)		Experimental Group (*n* = 26)		*p*-Value
	*Mean*	*SD*	*Mean*	*SD*	
**Age (years)**	21	1.72	20.96	1.68	0.935
**High (cm)**	167	0.08	167	0.07	0.944
**Weight (Kg)**	70.6	11.47	67.8	12.31	0.397
**CVA static (°)**	46.99	2.38	45.92	3.11	0.168
**CVA dynamic (*°*)**	45.89	2.69	44.84	3.94	0.269
**CFT (mmHg)**	26.38	3.21	25.38	2.45	0.212
**Current Pain (cm)**	0.72	1.16	0.47	0.99	0.402
**Usual pain (cm)**	1.7	2.40	1.03	1.90	0.220
**Discomfort Head (cm)**	1.39	2.34	0.71	1.58	0.226
**Discomfort Neck (cm)**	1.86	2.20	1.70	1.93	0.785
**Discomfort Shoulders and upper arms (cm)**	1.04	1.25	1.38	2.04	0.474
**Discomfort Middle back (cm)**	0.67	1.26	0.93	1.75	0.547
**Discomfort Lower back (cm)**	1.23	1.69	1.14	2.07	0.867
**Discomfort Forearms (cm)**	0.81	2.41	0.49	1.63	0.570
**Discomfort Wrists/hands (cm)**	0.92	2.29	0.26	2.01	0.508

**Table 3:** Post hoc comparisons in craniovertebral angle and endurance of deep neck flexors between groups.

Outcome Measures	Change B–C		Change C–D		Change B–D	
	*Mean*	*SD*	*Mean*	*SD*	*Mean*	*SD*
CVA_static_ (°)-CG	0.3238	2.32	−0.0551	0.43	0.2687	2.08
CVA_static_ (°)-IG	7.5431	2.13	0.5595	1.63	8.1026	2.58
***p*** **-value**	<0.01		0.070		<0.01	
**Effect size (r)**	0.73		0.06		0.74	
CVA_dynamic_ (°)-CG	−0.4497	2.37	0.1591	0.48	−0.2906	2.29
CVA_dynamic_ (°)-IG	5.1349	4.15	0.0282	1.86	5.1631	4.88
***p*** **-value**	<0.01		0.732		<0.01	
**Effect size (r)**	0.41		0.002		0.34	
CFT (mmHg)-CG	0.6154	2.57	−0.0769	0.39	0.5385	0.21
CFT (mmHg)-IG	2.9231	2.13	0.8462	1.89	3.7692	2.57
***p*** **-value**	<0.01		0.022		<0.01	
**Effect size (r)**	0.19		0.10		0.32	

## Data Availability

Data is unavailable due to privacy or ethical restrictions.

## Supplementary Materials

Supporting information can be downloaded at: https://www.mdpi.com/article/10.3390/brainsci15080873/s1, Table S1: CONSORT 2025 checklist item description.

## Abbreviations

The following abbreviations are used in this manuscript:

CFT	Craniocervical Flexion Test
CG	Control Group
CI	Confidence Intervals
CMDQ	Cornell Musculoskeletal Discomfort Questionnaire
CVA	Craniovertebral Angle
FHP	Forward Head Posture
IG	Intervention Group
MD	Mean Difference
MCTE	Motor Control Therapeutic Exercise
RSES	Rosenberg Sorensen Self-Esteem Scale
SD	Standard Deviation
SDD%	Smallest Detectable Difference (Percentage)
SEM	Standard Error of Measurement
VR	Virtual Reality

## Appendix A

This supplementary video complements the main manuscript by providing a clear, visual breakdown of the study’s two cohorts.

Video link: https://www.youtube.com/watch?v=Ws8WW-evLF0&feature=youtu.be (available from 2 June 2019).
